# What is worth knowing about peritoneal metastases in colorectal cancer?

**DOI:** 10.3389/fsurg.2025.1719153

**Published:** 2026-01-14

**Authors:** Michał Stańczak, Wiesław Kruszewski, Maciej Ciesielski, Jakub Walczak, Piotr Kurek, Tomasz Buczek, Mariusz Szajewski

**Affiliations:** 1 Oncological Surgery Department, Medical University of Gdansk, Gdańsk, Poland; 2 Department of Surgical Oncology with the Subunit of Breast Cancer, Skin and Soft Tissue Surgery, Maritime Hospital PCK, Gdynia, Poland

**Keywords:** colorectal cancer, peritoneal metastasis, cytoreductive surgery, HIPEC, PIPAC

## Abstract

Peritoneal metastases (PM) from colorectal cancer (CRC) represent a unique clinical challenge with distinct biological behavior and therapeutic implications. Although PM has traditionally been associated with poor prognosis, recent advances in diagnostics, surgical techniques, and intraperitoneal therapies have offered selected patients opportunities for prolonged survival and, in some cases, long-term disease-free status. This review discusses the pathophysiology, risk factors, diagnostic strategies, and treatment options for CRC patients with PM. The peritoneum is the second most common site of CRC metastasis after the liver, with synchronous and metachronous PM occurring at similar rates. Risk factors include right-sided colon tumors, BRAF mutations, and mucinous histology. Diagnosis relies on imaging modalities such as CT, MRI, PET/CT, and laparoscopy, although sensitivity remains limited for small-volume disease. The peritoneal carcinomatosis index (PCI) is a critical prognostic and therapeutic decision-making tool. Cytoreductive surgery (CRS) with or without hyperthermic intraperitoneal chemotherapy (HIPEC) has been shown to improve survival, particularly in well-selected patients with limited PCI and resectable disease. While the role of HIPEC remains debated following the PRODIGE 7 trial, alternative approaches such as pressurized intraperitoneal aerosol chemotherapy (PIPAC) are emerging. Systemic chemotherapy remains foundational, but response in PM can differ from parenchymal metastases due to the peritoneal–plasma barrier. Overall, outcomes are most favorable when patients are managed in specialized centers by multidisciplinary teams offering individualized, biomarker-informed treatment strategies. Ongoing research into molecular predictors and innovative drug delivery methods is critical to further improving prognosis for this complex patient population.

## Introduction

Colorectal cancer (CRC) is the third most common cancer in the world and is the second leading cause of cancer-related deaths (1). Metastasis of CRC to the parenchymal organs, especially when occurring in an isolated form, does not preclude recovery from the disease. Peritoneal metastases (PM), although still considered a sign of an incurable form of CRC, in selected cases allow for cure or a significant increase in survival. As early as 2001, Paul H. Sugarbaker (2), a pioneer in the treatment of PM from CRC, indicated that favorable outcomes are achievable when PM are limited in extent and can be completely or nearly completely resected, followed by hyperthermia-induced intraperitoneal chemotherapy (HIPEC) and systemic therapy.

In 2003, Verwaal et al. (3) published the results of a phase III randomized trial inspired by the hypothesis that, at an early stage of development, PM from CRC may represent the sole site of metastatic disease. This hypothesis was also supported by findings from earlier uncontrolled studies suggesting that cytoreductive surgery (CRS) combined with HIPEC offers superior outcomes compared with standard systemic chemotherapy.

The results of the study, with a median follow-up of 21.6 months, demonstrated significantly prolonged survival (median 22.3 months) in patients who underwent CRS plus HIPEC followed by systemic chemotherapy, compared with patients treated with systemic chemotherapy alone (median 12.6 months, *p* = 0.032).

A strong positive correlation was also observed between improved prognosis and both the completeness of cytoreduction (log-rank test, *p* < 0.0001) and a lower number of peritoneal regions involved by metastases (log-rank test, *p* < 0.0001) (3).

After an additional 5 years of follow-up in the same patient cohort, these findings were confirmed, showing a median disease-specific survival of 22.2 months in the CRS plus HIPEC arm vs. 12.6 months in the non-HIPEC arm (*p* = 0.028) and a 5-year survival rate of 45% among patients who achieved R1 cytoreduction (4).

In 2022, Tonello et al. (5) reported similar results, demonstrating a 5-year survival rate of 42.3% among 437 CRC patients who underwent CRS plus HIPEC. In contrast, the median survival time for untreated patients with peritoneal metastases is approximately 5 months (6).

In the American intergroup for colorectal cancer tumor-node-metastasis (AICC TNM) classification for colorectal cancer, the presence of a single organ metastasis was designated as M1a, multiple organ metastasis as M1b, and peritoneal metastasis as the worst prognostic feature M1c (7). Indeed, PM in CRC have a more deleterious impact on prognosis than metastases to the parenchymal organs, such as the liver or lungs. It was clearly shown in the study by Franko et al. (8) analyzing the material of 10,533 not previously treated patients enrolled in phase III randomized trials. As it was stated in the conclusions of the study, PM are associated with a worse prognosis, whether isolated or in combination with other metastatic locations. It has been noted that PM are a distinct disease entity, representing a promising target for new therapeutic strategies for CRC (8–11). To date, no clear consensus has been established regarding the optimal diagnostic and therapeutic management of patients with CRC and PM (12, 13). The European Society for Medical Oncology (ESMO) currently recommends restricting the use of hyperthermic intraperitoneal chemotherapy (HIPEC) to clinical trial settings only (14, 15). Given that the management of CRC patients with PM remains an open and challenging issue for surgical oncologists, we aimed to review and summarize the current state of knowledge on this topic.

## Methods

This narrative literature review aims to provide a comprehensive overview of existing knowledge on the biology of CRC with PM and of the currently available optimal diagnostic and therapeutic approaches for patients with CRC and PM.

English-language medical literature published up to 24 October 2025, and covering the last 5 years, was searched in the PubMed database. The keywords “colorectal cancer” and “peritoneal metastasis,” combined with the Boolean operator AND, were used for the search. Systematic, scoping, and narrative reviews, letters to the editor, and original research articles from specialized centers in peritoneal malignancy and CRC-PM treatment and from molecular biology units were screened and included.

## Epidemiology of peritoneal metastasis in CRC

After the liver, the peritoneum represents the second most common site of metastatic spread in CRC. PM are detected synchronously in approximately 4%–7.5% of patients undergoing surgery, despite their absence in preoperative imaging or staging assessments (16–18). In approximately 2%–4% of surgically treated CRC patients, PM constitute the only site of metastatic disease (8, 16, 17, 19).

Metachronous peritoneal metastases occur with a frequency similar to that of synchronous metastases and are observed in >4% of CRC patients (16, 18–21). Among patients who die from CRC, PM can be found in up to 80% of cases (22).

Peritoneal metastases are more common in colon cancer than in rectal cancer, particularly in right-sided primary tumors. In more advanced lesions—more frequently located in the right colon—serosal invasion facilitates the dissemination of malignant cells into the peritoneal cavity. In contrast, rectal cancers tend to infiltrate the retroperitoneal space rather than the peritoneum (17–19).

In the absence of a consensus on the definition of metachronous metastases, it is generally accepted that PM are metastases not recognized at the time of the first operation but recognized, for example, after 3 or 6 months (16, 20). In an analysis of the material from the Netherlands Cancer Registry of 7,233 patients with CRC, PM were considered synchronous up to 90 days after surgery or from the establishment of the diagnosis in those who were not operated. The remainder is considered metachronous. Synchronous metastases were detected in 5.7% of patients, of whom 3.4% had metastases to both the peritoneum and parenchymal organs and 2.3% had metastases only to the peritoneum. Among the 5,860 operated patients without synchronous peritoneal metastases (but with or without metastases to the parenchymal organs), metachronous peritoneal metastases were revealed in 5.6% of cases with a median time of approximately 15 months and a follow-up time of up to 38.4 months. In a group of 485 patients with synchronous metastases to the parenchymal organs who underwent surgery with the intention of a cure, metachronous metastases to the peritoneum occurred in almost 17% of patients, with metachronous metastases to the peritoneum and parenchymal organs in approximately 15% and metachronous metastases to the peritoneum only in approximately 2%. In contrast, in a group of 5,375 radically operated patients without synchronous metastases, metachronous metastases only to the peritoneum and metastases to both the peritoneum and parenchymal organs were diagnosed in 1.6% and 3% of patients, respectively. The authors emphasized that compared with other reports, these are the highest published rates of peritoneal metastases in similar material (16).

## Genesis of peritoneal metastases

The phenomenon of cancer cell detachment from the primary tumor and their multistep journey toward metastatic colonization sites remains the subject of intensive research, as each stage of this process may serve as a potential therapeutic target in the fight against malignancy. There are three principal routes of CRC dissemination: hematogenous, lymphatic, and transcoelomic.

In the case of PM, tumor cells may directly exfoliate from the surface of primary lesions that invade through the full thickness of the bowel wall and reach the serosal layer. Downregulation of specific adhesion molecules, such as E-cadherin, promotes this phenomenon.

High interstitial fluid pressure within the primary tumor—resulting from osmotic gradients induced by anaerobic glycolysis, rapid cellular proliferation, hypoperfusion, increased vascular permeability, and ineffective lymphatic drainage accompanied by stromal fibrosis—further facilitates the detachment of cancer cells from the primary site.

Surgical intervention may also contribute to an increased risk of intraperitoneal dissemination of tumor cells, either from the exposed tumor surface or through the opening of blood and/or lymphatic vessels during the procedure (10, 22–24).

Cancer cells can additionally reach the peritoneum via lymphatic pathways—massive nodal involvement being a recognized risk factor for PM even in non-serosa-invasive CRC—or through hematogenous spread (10, 22).

Diaphragmatic excursion, as well as gravity and the anatomy of the mesenteric intestine and peritoneum, promotes the peritoneal fluid flowing clockwise to the pelvis and from the pelvis along the paracolic gutter on the right toward the subdiaphragmatic space. This promotes tumor cell colonization in the distant peritoneum like the rectovesical pouch in men and the cavity of Douglas in women, the right lower quadrant in the terminal mesenteric area of the small intestine, the left lower quadrant along the upper edge of the mesenteric sigmoid colon and descending colon parenterally lateral to the cecum and ascending colon, the posterior right subhepatic space, and the right subphrenic space. The greater omentum is also a common location for PM (22–24).

Tissue factor (TF) initiates an extrinsic cascade in the coagulation system and is encoded by *F3*, a 12.5 kb gene located on chromosome 1p21.3 (25). CRC cells have been shown to express TF (26). Detached from the tumor surface, they activate the extrinsic cascade via TF, as in the coagulation system, leading to the production of fibrin fibers that enable them to fuse into polyclonal groupings of cells. Involving the same mechanism as in the formation of the cell grouping, they attach to the mesothelium covering the peritoneum. Cancer cell clustering may help protect the inner cells from immune attack (27). As demonstrated by Miyazaki et al. (27), each peritoneal metastasis is derived from at least tens to hundreds of clustered cancer cells. The multiclonal cancer cell clusters are the source of polyclonal PM. The process of cancer cell clustering may be enhanced by the procoagulant activity of ascites. In the research by Miyazaki et al. (27), they added cleared ascites derived from a cancer patient to the cancer cells in suspension culture, which induced extensive cancer cell clustering. Fractionation of ascites by centrifugation revealed that soluble components, rather than cellular or insoluble components, induced cell clustering. The polyclonal nature of PM makes them similar to those observed in the liver and lymph nodes, which are also the result of polyclonal seeding from the primary tumors. Cai et al. (28) showed that the liver and lymph metastases are derived from multiclonal clusters of circulating tumor cells released from the primary tumor. They sequenced the exomes of 150 single cells from primary tumor, liver metastasis, lymphatic metastasis, and an independent adenoma from a late-stage colon cancer patient. They revealed that lymphatic and liver metastases originated from the same region of the primary tumor and that the liver metastasis was derived directly from the primary tumor, bypassing the lymph nodes. The genetic heterogeneity of metastases was greater than that of the primary tumor, which indicates that they arose from clusters of circulating tumor cells of polyclonal origin (28).

The activation of the epithelial–mesenchymal transition (EMT) in carcinoma cells is responsible for their detachment from the primary tumor and the acquisition of invasive and migratory capabilities that enable dissemination to distant sites (29). These cells adopt a spindle-shaped morphology, which renders them more invasive. By forming multicellular aggregates, or “spheroids,” composed not only of cancer cells but also stromal and immune cells (including neutrophils), they actively remodel the microenvironment in a manner that promotes further tumor progression. They remain in this spheroid form until disaggregation occurs at peritoneal sites predisposed to metastatic implantation (22, 24, 29).

Adhesion molecules mediating cluster formation, such as specific integrins, confer resistance to anoikis—a form of apoptosis induced by loss of cell–matrix interaction. The multiclonal nature of these clusters may also facilitate protection from the host immune response, enhancing their survival and metastatic potential (10, 22, 24).

On the peritoneum, particularly on the greater omentum, are located submesothelial secondary lymphoid organs as organized aggregates of immune cells clustering with dense capillary networks called milky spots (10, 22, 24). Milky spots present particularly high levels of adhesion molecules that facilitate adhesion to tumor cells. Abundance of tissue-resident macrophages, a typical feature of the peritoneal cavity, differentiates in milky spots and contributes to the formation of an immunosuppressive niche during PM formation, preventing cytotoxic T cells from killing cancer cells (10).

Another way to facilitate EMT and adhesion of cancer cells to the omental mesothelium is through a web-like filamentous extracellular structure of DNA, histones, and cytotoxic granule-derived proteins extruded by activated neutrophils. They form a premetastatic niche called neutrophil extracellular traps (NETs). Neutrophils play critical roles in the eradication of pathogens and in cancer initiation and progression. Tumor-infiltrating neutrophils may be reprogrammed, for example, by TGFβ. NETs can be easily induced by tumor-derived exosomes or by elements of the tumor microenvironment (TME) like cancer-associated fibroblasts (CAFs) and platelets. Activated by tumor platelets, neutrophils promote intravascular NET formation. Experiencing hypoxia, cancer cells possess an enhanced ability to induce NETs. Key NET components affecting cancer progression include enzymes eliciting cytotoxic and proteolytic effects. One of the neutrophil-produced metalloproteinases triggers an “angiogenic switch” during cancer progression. NET-bound cathepsin G, by cleaving and activating certain metalloproteases, enables cell invasion by proteolyzing extracellular matrix (ECM) components. NETs can be critical components of different microenvironments that favor cancer initiation and can promote metabolic changes within the TME. It is suggested that NETs promote cancer development in the primary tumor niche. Actively regulating immune cell function, NETs can establish an immunosuppressive niche. NET–DNA structure may act as a physical barrier that limits contact between cancer cells and natural killer and T cells. Adaptive anticancer immune responses result in T-cell exhaustion and dysfunction in TME, thanks to immunomodulatory PDL-1 embedded within NETs. NETs can stimulate differentiation of immunosuppressive regulatory T cells. The upregulation and secretion of growth factors and other ligands that support the recruitment of immunosuppressive cells in tumor stroma are associated with the mesenchymal state. NETs have been found to be critical for establishing metastases (29, 30).

Adhesion-regulating proteins are required to attach the tumor cells to the mesothelium. Among others, glycosaminoglycans on mesothelial cells, integrins on tumor cells, and factors of peritoneal ECM such as fibronectin, laminin, and collagens take part in this process. Successful attachment is followed by angiogenesis (10).

Epithelial mesothelial cells contribute to the creation of a tumor-promoting niche by transitioning to CAFs via mesothelial–mesenchymal transition (MMT) and migrating into the stroma. MMT is actively induced by cancer cells (10). Tumor growth factor β (TGFβ) is recognized as a major inducer of the MMT (31). CAFs secrete oncogenic transcription factors into cancer cells and activate other mechanisms that facilitate cancer cells to modify the surrounding microenvironment for growth (10, 22, 32). Successful modification of the peritoneal microenvironment allows metastatic cancer cells to migrate through the mesothelium into the submesothelium and give rise to PM. The process of nesting of the peritoneal adherent cancer cell complex is accompanied by angiogenesis (10, 22, 27).

The predominant immune cell population within the peritoneal cavity consists of macrophages, which represent a highly heterogeneous group. Milky spots serve as an important reservoir for peritoneal macrophages. These macrophages participate in the formation of multicellular spheroids, acting as integral components of tumor cell clusters.

A key role in promoting tumor cell survival within the peritoneal cavity and in the establishment of metastatic foci is played by subpopulations of peritoneal macrophages that differentiate into tumor-associated macrophages (TAMs). These TAMs protect cancer cells from immune surveillance and facilitate their survival and further progression.

Peritoneal macrophages activate multiple signaling pathways in cancer cells, concomitant with the upregulation of genes encoding extracellular matrix metalloproteinase inducers. TIM-4^+^ cavity-resident macrophages prevent cytotoxic T cells from eliminating cancer cells, thereby promoting metastatic dissemination. Similarly, GATA6^+^ peritoneal macrophages enhance the growth of metastases that have breached the visceral mesothelium through PD-L1-mediated immunosuppression. Moreover, peritoneal macrophages contribute to angiogenesis by secreting proangiogenic factors (10, 22, 32).

TAMs are a key component of the TME which promote angiogenesis, tumor metastasis, and immune evasion. In the peritoneal cavity, resident macrophages support tumor cell colonization and proliferation, and also suppress the anti-tumor immune response (32). TAMs are recognized as the dominant suppressive cells associated with immune evasion in the intratumoral microenvironment. They promote immune evasion through multiple routes. In the case of ovarian cancer, CD24 expression promotes immune evasion by interacting with the inhibitory receptor expressed on TAMs. Another example concerns the successful sequestration of CD8^+^ T cells away from tumor targets by resident macrophages (32).

## Factors that increase the risk of peritoneal metastasis

In molecular consensus molecular subtype (CMS) classification of CRC, four subtypes, CMS1–CMS4, are identified, each characterized by different prognoses. CMS4, called “mesenchymal subtype,” is associated with the worst prognosis of CRC patients, and most PM are observed in CMS4 tumors. Stemness, epithelial–mesenchymal transition, and extracellular matrix remodeling transcriptional signature characterize the CMS4 subtype. Upregulation of EMT pathways, TGFβ signaling, matrix remodeling, stromal infiltration, poor relapse-free and survival are features of CMS4 CRCs. Typically for CMS4, and also for CMS1, undifferentiated cells have a high expression of TGFβ-provoked genes containing TGFβ1/2 cytokines. Increased expression of TGFβ in CRC tumors gives them a high metastatic potential. TGFβ signaling in CAFs stimulates the initiating capacity for cancer cells. TGFβ1/2 paracrine signaling is responsible for the occurrence of premetastatic features of the CAFs, which indicates the modulating role of the tumor microenvironment in cancer cell expression (33).

In their publication, Mardiah et al. (34) cited reports indicating that the three groups of transcription factors, namely, *Snail*, Twist, and *ZEB1*, play a role in activating EMT. They provided evidence that canonical signaling path, *TGF-β*, induces Smad binding to promoters of various transcription factors of EMT regulators such as *Snail*, Slug, *Twist1*, and *ZEB1* and that the regulatory transcription factor EMT causes decreased expression of epithelial markers, namely, *E-cadherin* and B catenin, and increased mesenchymal markers, such as N-cadherin and vimentin. Previous studies have shown that the expression of certain genes, along with the redundancy of various transcription factors regulating EMT, is associated with invasion, metastasis, and poor prognosis in patients with colorectal cancer. However, they also presented the results of their study, which has led them to speculate that the alterations in gene expression related to EMT within the *TGF-β/Smad* pathway in metastatic colorectal cancer are likely associated with the partial processes of EMT and mesenchymal–epithelial transition (MET) and that these alterations may contribute to further metastatic potential and increase the malignancy of the cancer (34). Metastasis in colorectal cancer is the toughest challenge to the success of treatment. The need to find biomarker candidates for metastasis and prognosis of colorectal cancer is increasingly important (24). Transcription factors belong to the predominant regulatory networks that contribute to differences in gene expression between molecular CRC subtypes (33). Nuclear factor Y (NF-Y), as a transcription factor, activates genes of cellular pathways commonly altered in cancer cells. As it was shown by Rigillo et al. (35), the NF-YA1 transcript in comparison with NF-YAs is higher in aggressive mesenchymal CRCs and predicts shorter patients' survival. Tumor cells with high NF-YA1 expression acquire enhanced single-cell migratory and invasive potential, probably as a consequence of low E-cadherin expression. E-cadherin serves as an adherens junction protein. Two isoforms of NF-YA control the expression of E-cadherin, with NF-YA activating and NF-YA1 inhibiting E-cadherin transcription. The results of Rigillo et al.’s (35) study also suggest that NF-YA1 expression predicts increased CRC aggressiveness and metastasis, presumably through the acquisition of a migratory/EMT phenotype typical for the CMS4 group.

## Impact of mutation pattern on metastatic spread and prognosis

Metastasis of CRC to the peritoneum is favored by mutations detected in primary tumor cells such as *BRAF* V600E or mutations in *Ras/Raf*, *PIK3/CA*, *PTEN*, *AKT1*, *SMAD2*, and *SMAD4.* In CRC, the most common mutated subtype is *KRAS* (50%–86%), followed by microsatellite instability *(MSI)* (10%–15%) and *BRAF* (10%). Mutations in KRAS and BRAF are generally mutually exclusive (5, 36). There is a significant concordance between the primary focus and PM as to the type of mutation (10, 37–39). The incorporation of mutation-specific data into tailoring the personalized way of treatment of CRC patients with PM may optimize local control of the disease, reduce the risk of peritoneal recurrence, and improve patients’ survival. Zucchini et al. (36) in their review on the impact of *RAS* and *BRAF* mutations and microsatellite status in PM from colorectal cancer after CRS + HIPEC stated that recognizing mutation subtypes may improve patient selection for treatment and the treatment results. They have noted that the impact of *KRAS*, *BRAF*, and *MSI* on outcomes in PM treated with CRS plus HIPEC remains underinvestigated. A review of recent reports indicated a negative impact of the *mKRAS* mutation on both disease-free survival (DFS) and overall survival (OS), as well as its association with an increased risk of peritoneal recurrence. The authors presented data analyzing *KRAS* and *BRAF* somatic variants, with *KRAS* *G12D* and *G12V* being the most common (39.7% and 19.1%, respectively) and *BRAF* *V600E* (80%) representing the predominant *BRAF* variant. They concluded that *BRAF V600E* and *KRAS G12V* mutations (*KRAS^MUT2^* profile) significantly decreased DFS. In multivariate analysis, the *G12V* variant was independently associated with poorer DFS (HR 2.63, *p* = 0.016). In the majority of analyzed studies by Zucchini et al. (36), BRAF mutation was identified as a negative prognostic factor. Some of the searched studies, while confirming the negative impact of mBRAF on overall survival, also indicated its effect on reducing DFS by BRAF V600 subtype compared with that observed in wild-type (WT) patients. Zucchini et al. have noticed that the impact of MSI on survival varied across the studies, with prevalence of those indicating better prognosis in MSI patients than in microsatellite stable (MSS) patients, especially if concomitant mutation in BRAF/RAS was present. In one of the analyzed studies, significantly better 5-year OS and DFS in the mBRAF/MSI group than in the mBRAF/MSS group was associated with synchronous peritoneal metastases and poorly differentiated tumors. In the analysis by Zucchini et al., the study by Tonello et al. (5) (*n* = 437 CRC patients treated with CRS plus HIPEC) provided evidence that the MSI status mitigates the negative effect of mKRAS/BRAF mutations on prognosis. A bivariate survival analysis of KRAS/BRAF mutation and MS status demonstrated an improved OS for the MSI patients in both the mutated and WT cases. The MSI and all-WT patients had a 5-year OS of 70.6% compared with 23.4% for the patients with MSS and KRAS/BRAF mutation. Similar survival was observed for the MSI patients with mutation and the MSS WT patients (5-year OS, 48.1%; *p* = 0.0002, log-rank). Analogous results were observed for DFS. Specifically, the MSI/all-WT patients had a 5-year DFS of 62.5% compared with 3.6% for the MSS/mutated patients (*p* = 0.00001, log-rank) (5). Zucchini et al. stated in their review that, given that MSI can attenuate the negative prognostic effect of KRAS/BRAF mutations, it should be considered a key stratification factor in patients undergoing CRS plus HIPEC (36).

In predictive models, independent risk factors for PM recurrently include tumor location in the right colon, depth of infiltration beyond the serous membrane (T4), and mucinous tumor differentiation (18, 40, 41). Tumors of the right colon grow asymptotically for a long time and are diagnosed at a more advanced stage (20, 37, 41). The CMS1 molecular subtype of CRC is most often right-sided, presents mainly in women with greater histopathological grade, and has poor survival after disease recurrence. *BRAF V600E* mutations are frequently found in CMS1 tumors and are related to the *MSI* phenotype. In CMS1 patients, the poorest prognostic impact of *BRAF* *V600E* was found in *MSS* tumors, and the OS rate was significantly lower than that in wild-type tumors. CMS1 includes a high percentage of CpG island methylator phenotype (CIMP)-high tumors. In CIMP-positive CRC promoter region of tumor suppressor genes is frequently hypermethylated, which causes a loss of function in these genes. The poor prognosis after disease recurrence is relevant for patients with *MSI* and *BRAF* mutations, collectively driving the tendency toward peritoneal metastasis (33).

Risk factors for PM diagnosed synchronously or metachronously also include the presence of signet-ring cells, involvement of surrounding lymph nodes (N1 and N2), and the presence of organ metastases (6, 10, 11, 16, 19, 22, 39, 41).

## Diagnosis of peritoneal metastases

The primary imaging study in the diagnosis of PM in the abdomen and pelvis is CT and MRI and PET/CT as indicated, especially to clarify the equivocal findings on CT/MRI or to rule out extraperitoneal disease. For all patients, CT of the chest is also mandatory (11, 41). The limited value of diagnosing peritoneal metastases with imaging studies is emphasized, as lesions <5 mm are often unnoticed. The detection rate on CT of nodules smaller than 5 mm is estimated to be 20%–25%. Involvement of the small bowel serosa is also difficult to detect on CT. MRI has higher sensitivity than CT. Superior soft tissue contrast resolution of MRI facilitates detection of deposits measuring <5 mm and those invading small bowel serosa, subphrenic peritoneum, and pelvic side wall. Invisible in CT, small pockets of mucinous fluid may be detected in an MRI scan. PET/CT is commonly used for the detection of unsuspected lymph node metastases and extraperitoneal metastases prior to planned CRS-HIPEC. While limited data are available regarding the accuracy of detecting the true extent of peritoneal disease on PET/CT, it is useful when CT findings are equivocal. Studies comparing PET/CT to the performance of MRI with diffusion sequences indicate that PET/CT appears to be complementary to, or more accurate than, MRI (42). It is recommended to combine evaluation on CT with MRI and with PET/CT (24, 43, 44).

Laparoscopy, including diagnostic laparoscopy, is an important invasive method for diagnosing PM. It is a less precise procedure in the evaluation of peritoneal carcinomatosis index (PCI) than the more invasive open surgery (40, 45). The effect of varying pressures generated during laparoscopy on increasing the risk of abdominal recurrence, including PM, has not been demonstrated (24). Addition of narrowband imaging (NBI) to laparoscopy may improve detection of low-burden PM. NBI is not useful during open surgery (45). When PM are detected during surgical exploration, the extent and resectability of the tumor lesions must be accurately assessed, and tissue samples must be taken from solid lesions and peritoneal fluid for cytologic evaluation. Surgical interventions should be limited to those necessary (e.g., removal of a bleeding tumor and decompressive fistula in gastrointestinal obstruction) before transferring the patient to a center with experience in treating these conditions (11, 41). Diagnostic removal of nodules and knowledge of the histologic type of PM can facilitate treatment planning (36, 46). Assessing for microsatellite instability, *RAS* and *BRAF* mutations, *ERBB2* status, and tumor mutational burden should be conducted as standard molecular analysis in CRC patients with PM, as mutation-informed strategies allow for refining the therapy strategies (11, 14, 36, 41).

Repeat diagnostic laparoscopy after chemotherapy has been found to be useful in patient selection for conversion to CRS for initially unresectable colorectal metastases, and is generally reserved for CRC patients with PM with PCI ≤ 19–25 amenable to complete or near complete cytoreduction (11, 41, 47).

Currently, liquid biopsy searching the ctDNA level in plasma shows limited value in detecting and monitoring PM (11, 41). Aside from history and physical examination, the routine serum markers, such as CEA, CA19-9, and CA125, may be useful in PM recurrence recognition (11, 41, 48).

## PCI

Almost three decades ago, a method for assessing the extent of peritoneal cancer spread was proposed and remains in use today as the peritoneal cancer index (PCI) (6). In addition to PCI, the assessment of intra-abdominal tumor burden—primarily in ovarian cancer—can be performed using a CT-based Fagotti scoring system, which determines the predictive index value (PIV). The CT features used to establish the PIV score include omental cake, liver surface involvement, lesser omentum involvement, splenic involvement, parietal or peritoneal carcinomatosis, diaphragmatic disease, and bowel infiltration. Each CT feature is graded according to the presence of tumor nodules, their extent, or the largest diameter of the lesion. The total PIV score, obtained by summing the individual regional scores, ranges from 0 to 14. Each parameter is valued with a 0 if absent or 2 if present. A value of ≥8 is related to suboptimal surgery (49). To assess the histologic response of peritoneal metastatic disease to chemotherapy, the peritoneal regression grading score (PRGS) is used. PRGS is a four-tier grading system considering several specific characteristics of PM, in particular their frequent mucinous character. For the test, biopsy samples are obtained from four abdominal quadrants for histopathological evaluation. Grade 1 indicates a complete response (absence of tumor cells); Grade 2, a major response (predominant regression features with only a few residual tumor cells); Grade 3, a minor response (presence of regressive changes but predominance of viable tumor cells); and Grade 4, no response (viable tumor cells without any regressive features). The mean PRGS is calculated from all available biopsy specimens (50).

**Figure 1:**
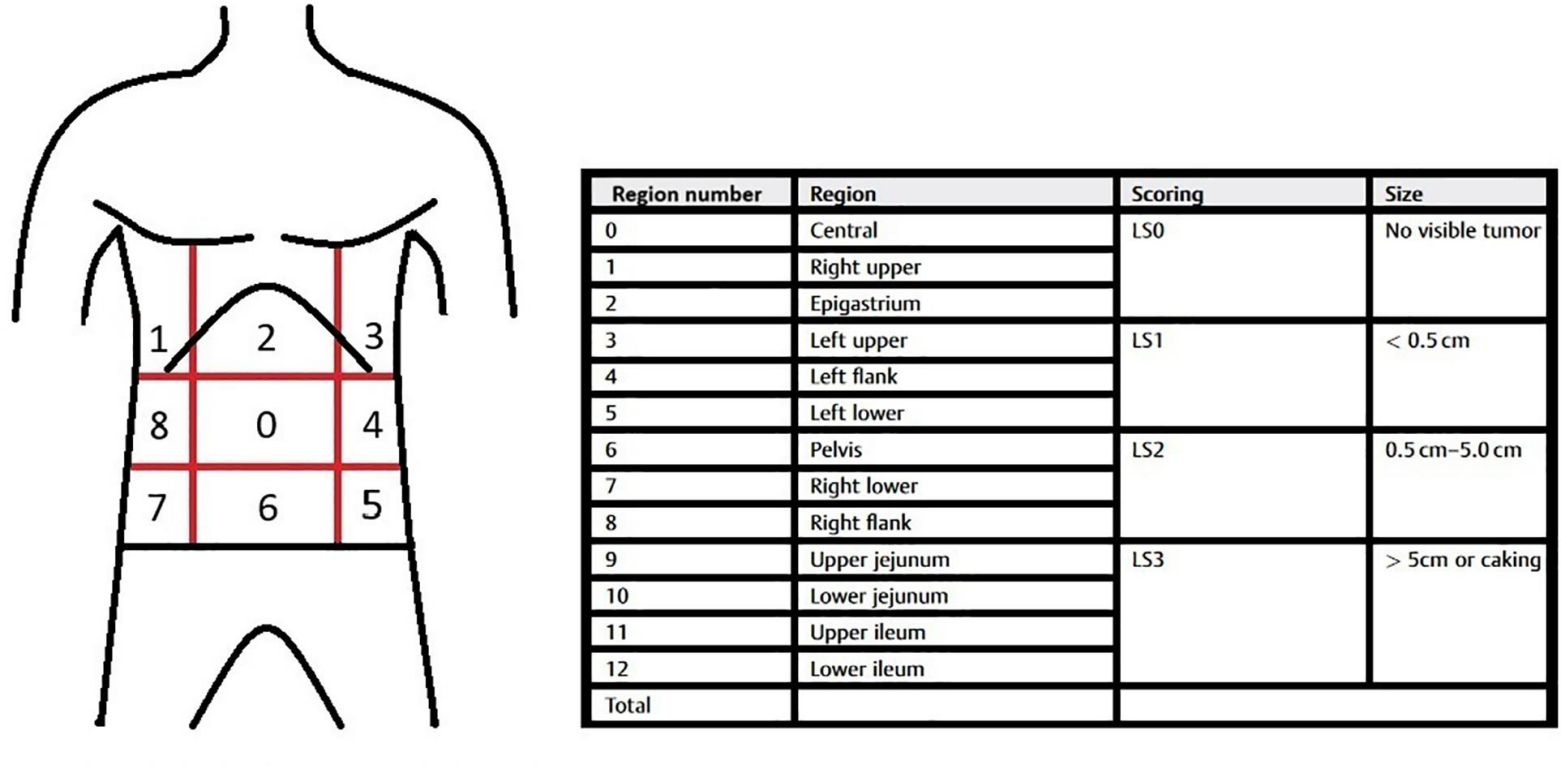
Regions of abdomen and lesion size score.

Surgeons should be familiar with the procedural requirements for assessing the PCI in CRC patients with peritoneal metastases, and all details regarding the extent of disease must be documented in the operative report. Whenever feasible, this documentation should be supplemented with photographic and/or video records. The abdominal cavity is divided into nine regions, with an additional four regions distinguished along the small intestine (Figure 1). Their exploration, taking into account the size of the metastatic lesions, is calculated into the number of points. The final PCI score is the sum of these numbers: 0 refers to the absence of metastases in a given area, 1 if they do not exceed 0.5 cm, 2 if they do not exceed 5 cm, and 3 for nodules over 5 cm or confluence (Table 1) (6).

**Table 1:** Peritoneal cancer index.

Region number	Region	Score	Scoring	Size
0	Central		LS0	No visible tumor
1	Right upper			
2	Epigastrium			
3	Left upper		LS1	<0.5 cm
4	Left flank			
5	Left lower			
6	Pelvis		LS2	0.5–5.0 cm
7	Right lower			
8	Right flank			
9	Upper jejunum		LS3	>5 cm or caking
10	Lower jejunum			
11	Upper ileum			
12	Lower ileum			
Total				

## Recommended treatment options for CRC patients with peritoneal metastases

Tonello et al. (13) analyzed current international guidelines regarding CRS and HIPEC as treatment modalities for patients with peritoneal metastases, including those originating from CRC. They noted substantial heterogeneity among the published recommendations and considerable variation in their implementation across different countries. However, a high level of consensus was observed in recommending CRS for resectable peritoneal metastases from CRC, although the strength of the recommendations varied. HIPEC was discussed only in 80.6% of guidelines, and recommended in 62,1% of them with the strengths consecutively: 6.9%, grade I; 31.1%, grade IIa; and 24.1%, grade IIb.

In August 2024, consensus on HIPEC on behalf of the Peritoneal Surface Oncology Group International (PSOGI) and French Network for Rare Peritoneal Malignancies (RENAPE) by Kusamura et al. (51) was published. As concerns HIPEC in the treatment of CRC with PM, there was a strong negative consensus regarding the short-duration, high-dose oxaliplatin protocol (55.7%), while a weak positive consensus (53.8%–64.3%) supported mitomycin C-based HIPEC, with the Dutch protocol being the preferred choice for both primary cytoreduction and recurrence. Determining the role of HIPEC after CRS 85.7% of panelists considered it essential.

In 2025, the Consensus Guideline for the Management of Colorectal Cancer with Peritoneal Metastases was issued as an update of the 2018 Chicago Consensus Guidelines under the auspices of the American Cancer Society (11). The authors underlined that given the low quality of existing evidence in the literature, recommendations were based primarily on expert opinion and focused on synchronous and metachronous PM of CRC. Early referral to the peritoneal surface malignancies (PSM) center was stressed by experts. In patients with synchronous metastases, systemic chemotherapy is a preferred initial treatment, and upfront CRS + intraperitoneal chemotherapy (IPCT) should only be considered in highly select patients. Right-sided tumors and signet-ring cell histology are the features of high risk of PM. If recurrence is detected after CRS ± IPCT, for both pathways, i.e., synchronous and metachronous PM, repeat CRS ± IPCT can be considered in selected patients. A weak recommendation was given for ctDNA testing during observation. The authors point out evidence from literature suggesting that CRS ± IPCT may benefit patients with limited extraperitoneal disease; however, they recommend the avoidance of major hepatectomy with CRS. Combined CRS with minor hepatectomy could be considered in select patients with limited liver metastases amenable to a CC0 resection and a PCI of *<*19 amenable to CC0-1. Krukenberg tumors do not preclude CRS ± IPCT. The authors further conclude that there remains limited evidence regarding optimal systemic therapy in patients with resectable CRC-PM. They emphasized that studies from the PSOGI Global Registry and the United States HIPEC Collaborative as well as several single-institution studies failed to demonstrate a benefit of neoadjuvant therapy. They also noticed that even proponents of neoadjuvant therapy argue for its potential to reduce PCI and increase complete cytoreduction, as it was not uniformly demonstrated across studies according to the authors. They referred to the CAIRO6 study, the first randomized controlled trial evaluating perioperative systemic therapy and CRS-HIPEC vs. CRS-HIPEC alone for resectable CRC-PM (NCT 02758951). A 38% major pathologic response rate among patients receiving neoadjuvant therapy has been observed. The results from the phase III randomized component of this trial remain unpublished. The consideration of upfront CRS ± IPCT is recommended for highly selected patients with complete cytoreduction predicted and presenting a high-performance status, low to moderate PCI, and low expected surgical morbidity. As the definitions of low or moderate PCI differ, the authors believe that extensive mesenteric deposits, small bowel deposits, or porta hepatis involvement might preclude complete cytoreduction and also that patients with poorly differentiated histology, such as signet-ring cell histology, should be treated with systemic therapy prior to CRS. After CRS, patients should receive adjuvant systemic therapy. Patients with no progression after systemic therapy and with a complete cytoreduction predicted should proceed with CRS ± IPCT. The authors refer to data from the PRODIGE 7 trial (5), emphasizing that even if they lack a survival benefit with oxaliplatin IPCT added to CRS, trial participants in both arms (CRS + IPCT vs. CRS) experienced a median overall survival of >44 months, which is higher than historical reports with systemic therapy alone. After CRS ± IPCT, additional systemic therapy should be considered. The authors unequivocally emphasize that the cornerstone of curative-intent treatment for CRC-PM remains CRS, with the objective of resecting all visible tumor implants within the peritoneal cavity. As concerns minimally invasive approaches for cytoreduction, they may be employed in selected patients with low PCI. According to the authors, the role of IPCT in treating PM of CRC remains contentious. The optimal drug dosing and duration remain uncertain, with some experts favoring mitomycin C for ≥ 90 min, an approach yet to be tested in RCTs. The authors referred to the results of randomized studies. HIPECT4 (52) demonstrated improved locoregional recurrence-free survival with surgical resection and prophylactic mitomycin C HIPEC compared with resection alone, while PROPHYLOCHIP (53) and COLOPEC (54) trials did not demonstrate benefit with prophylactic oxaliplatin HIPEC. Given the lack of unequivocal evidence supporting the benefit of IPCT, the authors recommend that decisions regarding its use should be made following thorough discussion between the patient and the multidisciplinary team (MDT). They also emphasize the uncertain role of systemic chemotherapy following CRS. While adjuvant chemotherapy is generally recommended for patients who have not received neoadjuvant treatment, its value in those previously exposed to neoadjuvant therapy, as well as in the perioperative setting, remains less clear.

Proponents of postoperative systemic chemotherapy highlight its potential role in preventing distant systemic relapse. In patients at risk of incomplete cytoreduction and without disease progression after chemotherapy, continuation of the previously administered systemic regimen is advised. In contrast, in cases of progression, second-line chemotherapy is recommended, with the authors underscoring that CRS ± IPCT should only be considered in carefully selected patients amenable to complete cytoreduction.

Besides considering best supportive care and potential enrollment in randomized clinical trials, pressurized intraperitoneal aerosol chemotherapy (PIPAC) is proposed as a therapeutic option, which should, however, be performed exclusively within the framework of clinical research.

In case of recurrence after CRS ± IPCT, additional systemic treatment is advocated, with repeat CRS ± IPCT being a potential option for some patients. The authors emphasized that, in line with their consensus, the PSOGI consensus (51) gave a conditional recommendation for HIPEC for patients with CRC-PM and recommended against short-duration and high-dose oxaliplatin. Both also recommended consideration of repeat CRS and IPCT for peritoneal recurrence at greater than 1 year after the index CRS. They also have noted that the 2023 ESMO Clinical Practice Guidelines for metastatic CRC (14, 15) recommend complete CRS and state that IPCT should only be offered in the setting of a clinical trial. They highlight the need for ongoing trials using other HIPEC regimens. Guidelines from PSOGI, American Society of Clinical Oncology (ASCO), ESMO, and the authors’ group stress the importance of multidisciplinary tumor boards and appropriate referral to PSM centers for PM from CRC.

A highly illustrated guideline proposed by Bhatt et al. (55), dedicated to surgeons, addresses the peritonectomy procedure and was developed due to existing inconsistencies in terminology and definitions related to peritonectomy techniques. The proposed classification system is complex, with multiple subdivisions, and is recommended for use—alongside the PCI—by all surgeons managing peritoneal malignancies. The success of cytoreductive surgery requires careful selection of patients for this procedure (56). The accepted cutoff point for PCI as to the curability of a patient with PM in CRC patients is up to 16 (11, 57). For optimal CRS, CC0 refers to the removal of all visible tumor foci from the peritoneum, and CC 1 defines the condition after complete removal of foci greater than 2.5 mm from the peritoneum. Suboptimal CRS refers to the condition when left peritoneal nodules measure 0.25–2.5 cm (CC 2) and when the left nodule size exceeds 2.5 cm or layered disease (CC 3) (56). Complete cytoreduction CC0 means that only microscopic residual disease was left potentially (2). Resection margin (R score) is also defined: R0, microscopically clean margins and no tumor cells in the peritoneal washings; R1, the presence of tumor cells in the peritoneal washings with macroscopically clean margins; R2a, nodules measuring ≤0.5 cm; R2b, nodules measuring >0.5 cm but smaller than 2 cm; and R2c, nodules measuring >2 cm. The optimal resection means CC0/CC1; R0/R1/R2a (2, 6).

Minor hepatectomy with CRS of PM is advised in select patients with limited liver metastases amenable to CC0 resection, and while PCI <19 amenable to CC0–1, the optimal patient population for a combined procedure remains unclear (11, 41, 58, 59). There is no longer a rule of up to three isolated liver metastases as an indication for their excision during CRS, but rather individual patient evaluation (11, 59, 60). The COLLISION trial (61) directly compared surgical resection for small-sized colorectal liver metastases (≤3 cm) with their ablation, and it was stopped early as it demonstrated a high likelihood of proving non-inferiority regarding OS, non-inferior local control, and fewer complications with thermal ablation compared with surgical resection. This may change the attitude to liver metastases management in patients suitable for CRS.

In CRC patients with PM, the main systemic chemotherapy medications are 5-FU with oxaliplatin and irinotecan, enriched with other drugs if indicated (biological and/or targeted agents, immunotherapy drugs, according to the individual molecular status). Biological agents, such as the anti-EGFR monoclonal antibodies cetuximab/panitumumab and the anti-VEGF monoclonal antibodies bevacizumab and ramucirumab, or anti-VEGF aflibercept, are added to cytotoxic drugs to enhance their efficacy. A combination of encorafenib plus cetuximab targets *BRAF V600E*-mutated tumors. Programmed death receptor-1 (PD-1)-blocking antibodies, such as pembrolizumab and nivolumab, as well as larotrectinib and entrectinib, indicated in tumors displaying the *NTRK* gene fusion, are also used in metastatic CRC (11, 14, 33, 41, 62)

As it was concluded in the review by Nikanjam and Kurzrock (63), resistance to oncology therapeutic agents occurs through clonal evolution, as metastatic cancers are heterogeneous within different sites in an individual and even within a single tumor. The authors emphasized that additional clonal evolution and molecular heterogeneity occur in response to therapy. According to their findings, supported by illustrative examples, identifying a molecular driver, selecting therapeutic agents that specifically target this driver, and initiating treatment early—at the time of diagnosis—before the development of a more complex molecular landscape, should provide optimal benefit for the patient. As many cancers are detected as advanced or metastatic disease, significant clonal evolution has occurred. Understanding the key genomic drivers for these cancers and targeting these alterations with individualized combination therapies as first-line treatments rather than later lines of therapy has the theoretical potential to provide more durable responses by limiting further clonal evolution (63). This is why a positive response of a primary tumor to a specific systemic treatment does not mean that its peritoneal metastases will respond similarly (63, 64). Because the peritoneal–plasma barrier reduces effective drug transfer, intraperitoneal chemotherapy enables high local drug concentrations, enhancing that way therapeutic efficacy while limiting systemic toxicity. From the three main approaches to intraperitoneal chemotherapy currently used: catheter-based, pressurized intraperitoneal aerosol chemotherapy (PIPAC), and HIPEC, the latter two are the most used in CRC patients with PM (65). HIPEC usually uses mitomycin C in monotherapy or with other cytostatics, such as 5-FU, cisplatin, doxorubicin, and oxaliplatin in monotherapy or in combination with mitomycin C (11, 41, 57, 65–67). The cytostatics are heated to an average of approximately 42°C. They fill the peritoneum in a closed-coat technique or in an open-coat technique in a time ranging from an average of 30 min (e.g., oxaliplatin) to 90 min (the others) (67). There are promising studies conducted on mass-based response screening as a comprehensive drug susceptibility test to guide personalized HIPEC by selection of proper HIPEC regimens. Drugs being tested include mitomycin C, oxaliplatin, irinotecan, mitomycin C plus cisplatin, and melphalan (9).

A promising method of palliative treatment, in addition to the administration of cytostatics through a drain into the peritoneal cavity, is PIPAC. This procedure uses laparoscopy and the ability to achieve variable pressures in the peritoneal cavity to administer cytostatics, which promotes overcoming intraperitoneal hydrostatic resistance generated by interstitial fluid pressure to facilitate drug penetration (65). In a randomized study, after three times of induction chemotherapy with this method, states of complete as well as significant histological response to the administered cytostatics were demonstrated (68). In selected patients, this enables the use of CRS + HIPEC sequentially (11, 24, 64, 65, 68). Currently, the usefulness of other methods of intraperitoneal drug delivery to PM patients, such as PIPAC with hyperthermia, high-intensity ultrasound, or the use of polymer gels as carriers for cytostatics, is also being tested (24).

## Outcomes of patients with peritoneal metastases

The independent and most important prognostic factor for CRC with PM is PCI. It significantly outweighs other factors of unfavorable prognosis with PM such as low differentiation of the primary tumor and others (20). Mutations in the *BRAF* gene and in the *K-ras* gene are also recognized independent factors for the worse prognosis of CRC patients with PM (11, 14, 20, 37, 69, 70). In these patients, *MSI* occurring simultaneously seems to attenuate the negative influence of *BRAF*/*RAS* mutation on prognosis (5, 36).

A proven element in improving the outcome of CRC with PM is their complete removal (11, 41, 56, 62, 71). In the PRODIGE 7 trial, a median survival of 41.2 months was achieved in the group treated with systemic chemotherapy and CRS, but without HIPEC, compared with 41.7 months if HIPEC with oxaliplatin was used (log-rank *p* = 0.99) (21). The remarkably longer survival time after CRS plus systemic therapy than expected indicates the value of CRS itself The prognostic benefit of PM resection without HIPEC was demonstrated by Yoshida et al. (71). After analyzing a retrospective cohort comprising 257 CRC patients who underwent CRS plus HIPEC and 156 patients treated with isolated resection of peritoneal metastases without peritonectomy or HIPEC, the authors found no significant difference in overall survival between the groups (hazard ratio 1.27, 95% confidence interval). When comparing the CRS plus HIPEC group with the surgery-without-peritonectomy group, the median overall survival was 42.3 months vs. 35.0 months (HR 0.91, 95% CI 0.69–1.19, *p* = 0.48).

Similarly, it was shown in the study by Young et al. (62) based on a cohort of 88,593 metastatic colon patients containing peritoneal and organ metastases, subjected to systemic therapy and colectomy vs. colectomy with CRS vs. surgery alone. Median OS was longer in the CRS group (34.4 months) vs. colectomy (26.7 months) vs. no surgery (13.2 months) (*p* < 0.001). However, patients who received HIPEC had improved OS than non-HIPEC patients.

With CRS + HIPEC, the chance of permanent cure of highly select CRC patients with PM has not only been raised in up to 16% of cases but also significantly prolonged the survival time of patients condemned to palliative management reaching up to 40%–45% 5-year survival (18, 53, 66). Optimal benefit from CRS is achieved in patients whose peritoneal metastases have metachronously manifested >1 year of asymptomatic course with PCI < 19–25, with isolated peritoneal metastases allowing their complete surgical removal (CC0 or R0) (56, 66). In the synchronous presence of PM and liver metastases, the prognosis is worse than for PM alone (60).

After CRS, a better prognosis in terms of time to recurrence and overall survival is observed when no penetration of single cells into the space separating the nodule from the peritoneum is observed (pushing-type) compared with the prognosis when metastatic cells are present in this space (infiltrative-type). Different histological types of nodules can be observed even in a single patient (72).

It has been noted that ascites accompanying PM of CRC decreases the response rate to chemotherapy and to immunotherapy in the group of tumors most responsive to this type of treatment. Chia et al. (64) commented on the finding that in *MSI-H* CRC tumors sensitive to immune checkpoint inhibition, the best response for immune checkpoint inhibition was noticed in the group without ascites and PM, followed by those with PM without ascites, and the least expressed in patients with PM and ascites. The authors concluded that PMs may demonstrate resistance to the immune checkpoint inhibitors that arise either from factors pertaining to tumor characteristics, the immunosuppressive state of the peritoneal cavity, paracrine factors within malignant ascites, or tumor–peritoneum interactions.

## Conclusions

A lot of questions remain to be answered concerning CRC patients with PM. Undoubtedly, they should be referred to and managed in the center experienced in peritoneal malignancy treatment and providing access to optimal methods of diagnosis and treatment of this disease. MDT must discuss with the patient the optimal way of treatment taking into account the patient’s state and history, PCI, and pathological and molecular characteristics of malignancy. Highly personalized treatment with exactly recognized and successfully attacked targets is a future in the successful management of CRC patients with PM.

## Summary

Synchronous and metachronous PM occur with similar frequency in CRC patients, who are still candidates for further treatment. As PM of CRC are distinct disease entity, a special way of management should be offered to these patients in a center dedicated to peritoneal malignancy, and under the supervision of MDT. The aim should always be to create conditions for the patient to optimally remove PM along with the primary focus, as this prolongs the time to recurrence and survival, and in selected cases leads to a permanent cure of the disease. The main element of therapeutic success is personalization of treatment relying on patient state, history, PCI, and pathological and molecular markers estimated. It contains neoadjuvant and adjuvant chemotherapy, surgery, and different modes of intraperitoneal therapy. Main diagnostic tools to estimate PCI are CT, MRI, and PET/CT, laparoscopy, and open surgery. Surgeons must be familiar with the details required to estimate PCI and what to do in case of PM. The main systemic therapy medications are 5-FU, oxaliplatin, and irinotecan, enriched with other drugs as indicated. The preferred cytostatic for HIPEC is mitomycin C. We are obliged to monitor the guidelines concerning CRC patients with PM management and implement them into practice.
